# Editorial: The Role of Peptide Hormones in Insect Physiology, Biochemistry, and Molecular Biology Processes

**DOI:** 10.3389/fphys.2021.644907

**Published:** 2021-02-02

**Authors:** Dov Borovsky, Yonggyun Kim, Klaus H. Hoffmann

**Affiliations:** ^1^Department of Biochemistry and Molecular Genetics, University of Colorado Anschutz Medical Aurora, Aurora, CO, United States; ^2^Andong National University Andong, Andong, South Korea; ^3^University of Bayreuth Animal Ecology I, Bayreuth, Germany

**Keywords:** cellular immunity, venom proteins, insect midgut, cold stress, muscle contraction, pyrokinins, TMOF, hypertrehalosemic hormone

The study on the effect of the ectoparasitoid *Pachycrepoideus vindemmiae* on the cellular and humoral immunity in *Drosophila melanogaster* by Yang et al. was investigated at the pupal stage. The authors describe the cellular and humoral response to *P. vindemmiae* by pupal *D. melanogaster* using, *in vivo*, and *in vitro* studies showing that the Toll and Imd immune pathways are activated upon parasitization followed by the JAK/STAT pathway.

Venom proteins in the pupal ectoparasitoid *Pachycrepoideus vindemmiae* of *Drosophila* were analyzed by Yang et al. Expression studies show that 20 venom proteins are expressed 419-fold higher in tissues that produce venom. Relationships between venom proteins of other five species from three parasitoid families detected ancient orthologs in Pteromalidae. The authors assigned the venom proteins to orthologous groups showing that considerable interspecific variation of venom proteins is found in parasitoids using phylogenetic classification.

Neuropeptides and G-protein coupled receptors (GPCR) were studied in the red palm weevil by Zhang et al. They reported that the weevil causes damage to palm trees and induces highly tuned physiological and behavioral adaptability. The authors identified neuropeptide precursors and their cognate receptors in infected and uninfected tissues and identified 43 putative neuropeptides and 44 putative receptors. Using RT-qPCR analyses they showed that genes coding for several neuropeptides were expressed in various tissues but not in the nervous system suggesting that some of the peptides and their receptors may have other roles. Pathogens infections upregulated several of the neuropeptides and their receptors like tachykinin related peptide receptor.

Peptide hormones play an important role in insect midgut, they regulate growth, development, immunity, homeostasis and stress. Wu et al. reviewed the function and importance of these hormones in the insect midgut. The authors described that not only peptide hormones play an important role in the control of insect gut physiology, but several other hormones that are secreted from the corpus allatum, prothoracic gland and neural cells also have a role in inducing and secreting insects' digestive enzymes. These hormones also recognize pathogenic bacteria and may signal the gut cells to produce peptide hormones to control pathogens.

Neuropeptides act like important messenger molecules in neurotransmission. Marciniak et al. reported that FMRFamide-related peptides signal and regulate muscle contraction in tenebrionid beetles. These authors reported that neuropeptides that exhibit myotropic properties in insects also known as FMRFamide-like peptides (FaLPs) are regulating the contractile activities of the heart, ejaculatory duct, oviduct, and the hindgut in two beetle species. The authors identified *in silico* a putative receptor in both species. They showed that the putative receptors are expressed in various tissues including the visceral organs. Amino acid sequencing identified the receptor as G-protein coupled receptor. A synthetic receptor FaLP peptide was synthesized by the authors and was shown to have cardioactivity in one beetle and an inhibitory effect in a different beetle and myostimulatory effect on the visceral organs.

Pyrokinins are neuropeptides with myotropic, pheromonotropic, and melanotropic roles in several insects. In this study Lajevardi and Paluzze characterized pyrokinin1 and 2 receptors in the mosquito *Aedes aegypti* using an heterologous cell system. The authors measured the transcript level expression of these receptors in adults and found that PK1-R transcript is highest in the posterior hindgut (rectum), whereas PK2-R was highly expressed in the anterior hindgut (ileum) and in the reproductive organs. Immunoreactivity studies confirmed that PK1-R transcripts are found in the rectum and form the target of Pk1 regulating excretion.

Trypsin modulating oostatic factor (TMOF), a mosquito decapeptide that regulates blood digestion was cloned and expressed by Borovsky et al. in the methylotrophic yeast *Pichia pastoris*. Ten gene copies of TMOF were expressed by the engineered *Pichia* cells. The cells were analyzed by Southern, and Northern blotting followed by ELISA showing that the engineered cells (1.65 × 10^8^ and 8.27 × 10^7^ cells) synthesize 229 and 114 μM of TMOF, respectively, causing 100% mortality to larval mosquito. Therefore, engineered *P. pastoris* expressing TMOF could be used in the future to control mosquito larvae.

Insects are capable of surviving in arid to freezing climates. Lubawy et al. reviewed the role of insect neuroendocrine system during cold stress. These authors reported that neuropeptides and biogenic amines play a central role in regulating cold hardiness. Cold stress caused the release of chemical signals that control osmoregulation by various peptides such as capability peptides (CAPA), inotocin (ITC), ion transport peptide (ITP), diuretic hormones and calcitonin (CAL), tachykinin related peptides (TRPs) and peptides that are involved in mobilization of body reserves. The authors' review summarizes the current knowledge on the role of neuropeptides in cold stress response.

Two adipokinetic neuropeptide hormones (AKH) are synthesized by the corpora cardiaca of the Indian stick insect. Katali et al. reported that these neuropeptides increase the trehalose levels in the hemolymph if the insect is ligated between the head and thorax. They tested the potency of 19 AKH analogs on trehalose levels and change in the heartbeat rate with AKH analogs containing single amino acid substitution at different positions. Analogs that were modified at the termini did not show reduced activity as compared with control AKH. Shorter AKH peptides did not bind to the AKH receptor and substitutions of a single amino acid in most places of the native AKH abolished binding to the receptor. These results indicate that binding of AKH to its receptor is sequence specific.

Lipids are considered the primary storage molecules and essential energy source for insects during physiological events of reproduction, flight, starvation, and diapause. Lipid metabolism occurs is the fat body. The role of peptide hormones in insect metabolism was reviewed by Toprak describing the roles of peptide hormones in lipogenesis by adipokinetic hormone and brain insulin-like peptides (ILP2, 3, and 5). On the other hand, lipolysis is controlled by insulin-growth factor (ILP6), neuropeptide F, allatostatin-A, corazonin, leucokinin, tachykinins, and limostatin. Several neuropeptides that induce lipogenesis were also described. The functions of peptide hormones in lipid metabolism during reproduction, flight, diapause, starvation, infection and immunity were highlighted.

The roles of the *pyrokinin and capa* GPCRs genes in the stink bug *Halyomorpha halys* were reported by Ahn et al. These genes express multiple neuropeptides that were tested in cell-based assays and by RT-PCR. Ahn et al. showed that *H. halys* pyrokinin receptor-1 responds to pyrokinin 2 peptides. Whereas, *H. halys* pyrokinin receptor 2 responded to pyrokinin 1 type peptides that are similar to diapause hormone. The expression of the receptor genes in different tissues and life stages was determined by RT-PCR. The experiments described by Ahn et al. show that several of the peptides tested have specificities to 6 GPCRs and several are activated by pyrokinin 2 and pyrokinin 1 peptides.

## Author Contributions

All authors listed have made a substantial, direct and intellectual contribution to the work, and approved it for publication.

## Conflict of Interest

The authors declare that the research was conducted in the absence of any commercial or financial relationships that could be construed as a potential conflict of interest.

